# Anosognosia and avoidant coping do not impact work in early Huntington's disease

**DOI:** 10.1177/18796397251349114

**Published:** 2025-06-11

**Authors:** Kasper Frederik van der Zwaan, Raymund AC Roos, Susanne T de Bot

**Affiliations:** 1 Department of Neurology, LUMC, Leiden, The Netherlands

**Keywords:** Huntington's disease, work, anosognosia, coping skills, neuropsychology

## Abstract

**Background:**

Work plays a crucial role in life, contributing to financial stability and well-being. Huntington's disease (HD), a genetic neurodegenerative disorder, can significantly affect work capacity. Anosognosia (lack of awareness of impairments) and avoidant coping are common in HD but remain unexplored in relation to work outcomes.

**Objective:**

This study investigated the relationships between anosognosia, coping styles, and work capacity in individuals with pre-motor manifest and motor manifest HD.

**Methods:**

Utilizing the HD-Work dataset, we analyzed motor and cognitive functioning, coping styles, work capacity, and anosognosia in participants with pre-motor manifest and motor manifest HD (n = 117). Anosognosia was operationalized through expert rating, participant - proxy, and cognitive – performance discrepancies. Work capacity was measured using the occupation item of the Total Functional Capacity scale, and coping styles were assessed with the *Utrechtse Coping Lijst*.

**Results:**

Anosognosia was strongly associated with cognitive decline, while avoidant coping was less prevalent. Both anosognosia and avoidance coping were correlated with frontal behaviors but not with work capacity. A positive association between avoidant coping and anosognosia was found. The most common coping style used was passive coping. Participants did not often seek social comfort.

**Conclusions:**

The best predictor of anosognosia was cognitive decline. The positive association between avoidant coping and anosognosia suggested a potential misattribution of avoidant coping to anosognosia. This study emphasized the importance of recognizing avoidant and passive coping strategies in early-stage HD, as well as anosognosia in relation to cognitive decline, even though these factors do not directly impact work capacity.

## Introduction

Work is an important part of life. It provides financial security and contributes significantly to fulfilment, personal identity, connection with others, and overall quality of life.^1,2^ Chronic diseases can impact the ability to work and, therefore, the factors contributing to personal well-being. Financial stress can have a further detrimental effect on the psychology of individuals, especially when work cessation is involuntary.^3^

Huntington's disease (HD) is a genetic neurodegenerative disorder caused by a CAG-polyglutamine repeat expansion of ≥36 in the huntingtin (*HTT*) gene.^4,5^ HD is strongly associated with reduced work capacity and work cessation. Moreover, 61–65% of patients experience work-related changes as the first notable functional decline.^6^ The decrease in function is caused by the triad of HD symptoms: cognitive decline, psychiatric characteristics, and motor dysfunction.^5^ The added impact of an employee's unawareness or lack of acknowledgment of their dysfunction related to HD remains unexplored.

Anosognosia is a condition in which individuals exhibit limited insight into the extent of their own impairments and are unaware of them, observed in a spectrum of neurological disorders affecting the frontoparietal lobe, especially HD.^7,8^ Anosognosia in HD is a multi-faceted phenomenon wherein individuals may be unaware of certain symptoms (e.g., chorea) while retaining insight into others (e.g., cognitive decline or apathy), reflecting that awareness can vary across different symptom domains.^7,9^ Moreover, anosognosia is particularly prevalent in the advanced stages of HD,^10^ when patients face challenges on all functional fronts, i.e., maintaining employment, managing finances, driving, performing household tasks, and maintaining self-sufficiency in activities of daily living.^11^ Perhaps the most striking example of this phenomenon is the manifestation of anosognosia for a symptom as pivotal as chorea,^12^ where movements to the observer are undeniable but are not registered by the patient. This highlights the complex nature of this disorder and the many factors that may be involved in its development. Moreover, anosognosia may be a confounding factor when interpreting patient-reported outcomes in HD research and clinical care.^13^ The direct effect of anosognosia on work outcomes, especially in pre-motor manifest and early-stage expanded gene carriers, has not yet been studied.

Individuals exhibit varying responses to managing stress and distress, a phenomenon commonly called coping style. Coping styles have been the subject of numerous theoretical frameworks and are assessed through many questionnaires. Broadly, coping styles can be categorized into two groups: effective and maladaptive. However, the effectiveness of a particular coping strategy hinges on the nature of the stressor and the response chosen. People with HD have a wide range of negative emotions and thoughts about their illness. These emotions and thoughts are linked to how people with HD cope with their illness. For example, some people may cope by seeking social support from others, while others may try to avoid thinking about their illness altogether.^14^ Avoidant coping, typically characterized by active efforts to evade stressors that induce anxiety, is often viewed as an ineffective strategy. In the context of HD, the stressor, being the disease with its neurodegenerative and progressive characteristics, persists without resolution.^15^

Although the cause of avoidant coping, which is a deliberate choice to manage stress, differs from that of anosognosia avoidance, which stems from unconscious cognitive impairment, the outcomes are often identical. In both scenarios, there is a situation where observers notice signs and symptoms of HD even as the patient denies experiencing any of them. Previous research has suggested that the two constructs are connected.^9,16^ The current study explores the effects of these two specific (neuro)psychological factors on work capacity in both pre-motor manifest and motor manifest HD. Furthermore, it aims to shed light on the prevalence of avoidant coping, other coping strategies, and anosognosia in the early stages of HD.

## Methods

We used the HD-work dataset consisting of 117 adult HD-expanded gene carriers included at the Leiden University Medical Centre between April 2019 and December 2020. HD-work was a prospective study that aimed to identify predictors of work-related changes and work cessation beyond the clinical characteristics of HD. Participants had to be between 18 and 67 years of age and have a confirmed CAG-repeat length ≥36 to participate. They also needed to work at the time of inclusion or until at least two years before. The two years were based on the WIA (Work and Income according to Labor Capacity Act) which is a Dutch law that governs how individuals with work incapacity are supported in their income and rehabilitation back into the workforce. All participants were Dutch and residing in the Netherlands. Written informed consent was obtained from all participants. The HD-Work study was approved on 14 March 2019 by the Medische Ethiek Toetsing Commissie – Leiden, Den Haag, Delft (METC-LDD) (reference: NL67070.058.18). This study is cross-sectional, using the baseline data only.

### Huntington specific measurements

The Unified Huntington's Disease Rating Scale (UHDRS) Total Motor Score (TMS) was used to assess motor function.^17^ Participants were divided into pre-motor manifest and motor manifest HD based on UHDRS diagnostic confidence levels (DCL). Here, scores range from 0 to 4, indicating the certainty of clinical HD diagnosis based on motor symptoms. A score of ≤ 3 is considered pre-motor manifest, and a score of 4 is motor manifest. Working capacity was assessed by the occupation item of the Total Functioning Capacity's (TFC).^18^ Here, the scores range from 0–3, with 0 indicating an inability to work, 1 indicating marginal working capacity only, 2 indicating altered hours or responsibilities and 3 indicating working at a normal level. Other items than occupation were not assessed in this study. The CAG-Age Product (CAP) score was used as a disease burden score.^19^

### Cognitive assessments

To evaluate cognitive functioning, we used the following assessments commonly used in HD studies: Symbol Digits Modalities Test (SDMT),^20^ Verbal Fluency Category Test (VFCT),^21^ Stroop Color Naming Test (SCNT), Stroop Word Reading Test (SWRT), Stroop Interference Test (SIT),^22^ Trail Making Test A and B (TMT A&B),^23^ and Verbal Fluency Letter Test (VFLT).^21^ All are measures of executive functioning, that measure either psychomotor speed, working memory, attentional shift, inhibitory ability, or a combination of two or more. We calculated a composite cognition z-score for each participant, refer to Supplemental Figure 1 for the equation used.

### Coping questionnaire

The *Utrechtse Coping Lijst* (UCL) was used to determine the coping styles used by the participants. The UCL is a Dutch questionnaire with good validity and reliability, demonstrated by its strong internal consistency (Cronbach's alpha ranging from 0.67 to 0.84 across different subscales) and test-retest reliability (correlation coefficients between 0.55 and 0.74 over a seventeen-month period).^24^ It evaluated the following coping styles: active coping (scores range from 7–28), palliative response; i.e., reduction of the stressor by not addressing the stressor itself (e.g., alcohol (ab)use or excessive work-outs) (8–32), avoidance, i.e., ignoring or avoiding the stressor, (8–32), seeking social support;, (6–24), passive response; i.e., a resigned response possibly by feeling overwhelmed, (7–28), expression of emotions (3–12), and reassuring thoughts; i.e., positive self-talk and focusing on the positive side (5–20). The scores are normed by the test constructors and categorized as follows: very high (≥ 95^th^ percentile), high (> 80^th^, < 95^th^ percentile), average (> 20^th^, < 80^th^ percentile, low (> 5^th^, < 20^th^ percentile), and very low (≤ 5^th^ percentile).

### Operationalization of anosognosia

To assess anosognosia in our participants, we employed three evaluation methods described in the work of Ruijter et al.^25^ We classified individuals as having anosognosia if they met the following criteria: being clinically assessed as lacking insight by the physician or neuropsychologist, or displaying a discrepancy between the (lower) self-reported and (higher) proxy measurement on the FRSBE, or their (better) self-predicted performance and (worse) actual performance. First, we conducted a clinical expert rating, where participants’ insight was clinically and independently estimated by a physician (neurologist) and a neuropsychologist. In these expert ratings, anosognosia was defined as a clear lack of awareness or acknowledgment of clinically evident symptoms and their impact on daily functioning, as determined through clinical interviews (such as the TFC), and direct observation. Raters made a simple yes/no judgment regarding the presence of anosognosia. If there was any doubt, the participant was classified as without anosognosia by that rater. The clinical expert ratings were conducted by three physicians and four neuropsychologists (The first author, KZ, was one of the neuropsychological raters, and the last author, SB, was one of the physician raters). One physician and one neuropsychologist rated each participant. The distribution of ratings ended up being balanced such that each physician and neuropsychologist evaluated an equal number of participants with and without anosognosia.

Therefore, inter-rater variability was minimized. Second, we examined discrepancies between participant self-assessment and the assessment provided by a partner or close relative (proxy). Our study compared participant’ and proxy responses using the Frontal Systems Behavior Scale (FRSBE), encompassing three domains: apathy, disinhibition, and executive dysfunction.^26^ This questionnaire has been used before in discrepancy analysis, and the domains tested are explicitly frontal behaviors, which are a relatively early feature of HD.^26^ third, we compared participant performance with predicted performance by comparing executive functioning as evaluated through composite cognitive z-scores with FRSBE's executive dysfunction domain, as judged by the participants. In this case, higher scores on either of the FRSBEs indicate more frontal behaviors. In the two scenarios that compare proxy and patient ratings and patient ratings and cognitive performance, a discrepancy was defined as a difference of more than or equal to 1.96 standard deviations from the mean deviation score. Ultimately, we devised a ‘combined measure’ by incorporating all three criteria to classify participants as with behaviors consistent with anosognosia if they met anyone or combination of these criteria. This combined measure was formulated by synthesizing the individual evaluation methods and setting a threshold for classification. While de Ruijter et al. (2020) utilized similar evaluation methods, they did not develop a combined measure; this approach is unique to our study.

### Statistical analysis

We used R version 4.2.2 for statistical analyses. Prior to conducting any tests, we assessed the normality of the data distributions using Shapiro-Wilk tests. For normally distributed data, we employed independent t-tests; otherwise, we used non-parametric versions of these tests, such as the Mann-Whitney U test. We also verified that continuous data met the assumptions for linear regression, including homogeneity of variances using the Levene's test. In cases where the assumptions were violated, non-parametric tests were applied.

First, we analyzed the sample's demographic information and compared scores between pre-motor manifest and motor manifest participants. These group comparisons were performed using independent t-tests (for continuous variables) and chi-square tests (for categorical variables). Subsequently, we compared scores between participants with anosognosia and participants without anosognosia using t-tests (for continuous variables) and chi-squared tests (for categorical variables). We have used Pearson's correlation to obtain information on association between variables.

Following that, we delved into the analysis of coping styles, comparing them to Dutch norms.^24^ Dutch norms refer to the standardized scores obtained from the general Dutch population, which provide a benchmark to compare individual coping scores against. These norms help to understand where the participants’ coping styles fall in relation to the average scores of the Dutch population, allowing for meaningful interpretation of the data. A network analysis was employed to analyze the coping styles. This network analysis is presented as a visualization with nodes and edges. The threshold for visualizing the correlation was arbitrarily set at r = 0.1. Nodes represent different coping styles, whereas edges reflect the significant correlations between them.^27,28^ Univariate and multivariate linear regression models were used to predict the impact of avoidant coping and anosognosia on work outcomes.

## Results

### Demography

Our cohort (n = 117) primarily consisted of working and middle-aged individuals, with a higher proportion of pre-motor manifest participants (n = 84) than motor manifest participants (n = 33) (Table 1). See Supplemental Table 1 for information on missing values. On average, the pre-motor manifest participants were slightly younger (t = –4.12, p < 0.001), exhibited better cognitive and motor performances (i.e., SDMT (t = 7.24, p = 0.000), VFCT (t = 3.40, p = 0.001), SCNT (t = 7.34, p < 0.001), SWRT (t = 5.05, p < 0.001), SIT (t = 5.70, p = 0.000), TMTA (t = –4.18, p < 0.001), TMTB (t = –3.47, p = 0.002), VFLT (t = 4.71, p < 0.001)), and scored lower in avoidant coping (t = –2.36, p = 0.019) while scoring higher in active coping (t = 2.77, p = 0.008), as compared to the motor manifest participants. Motor manifest participants scored higher on the UHDRS-TMS compared to pre-motor manifest participants (t = –10.67, p < 0.001) and the CAP-score (t = –8.69, p < 0.001).

**Table 1.** Demographics of the complete sample and comparison of pre-motor and motor manifest participants.

	Total		Pre-motor manifest (N = 84)		Motor manifest (N = 33)			
	Mean (range)	SD	Mean (range)	SD	Mean (range)	SD	Δ	p
Demographical								
Age	44.1 (21–64)	10.5	41.8 (25–63)	10.0	50.0 (21–64)	9.5	8.0	**<0.001**
CAG-repeat length	42.4	2.9	41.7	2.4	44.1	3.3	2.6	**<0.001**
Sex (f/m (%))	51/66 (43.6/56.4)		38/46 (45.2/54.8)		13/20 (39.4/60.6)			0.714
CAP-score	82.7	22.3	73.2	17.3	105.0	17.5	31.6	**<0.001**
Anosognosia								
Physician (y/n (%))	13/91 (12.5/87.5)		7/66 (9.6/90.4)		6/25 (19.4/80.6)			0.292
Psychologist (y/n (%))	17/95 (15.2/84.8)		7/73 (8.8/91.2)		10/22 (31.3/68.7)			**0.007**
Participant/performance (y/n (%))	6/111 (5.1/94.9)		81/3 (3.6/96.4)		3/30 (9.1/90.9)			0.452
Participant/proxy (y/n (%))	5/112 (4.3/95.7)		81/3 (3.6/96.4)		2/31 (6.1/93.6)			0.927
Combined measure (y/n (%))	30/87 (25.6/74.4)		15/69 (17.9/82.1)		15/18 (45.5/54.5)			**0.004**
Cognition								
SDMT	49.4	12.5	53.9	10.5	38.6	9.9	−15.5	**<0.001**
VFCT	21.0	6.6	22.3	6.4	17.9	6.0	−4.7	**0.001**
SCNT	67.6	16	73.2	14.1	54.0	11.7	−19.3	**<0.001**
SWRT	89.5	21.2	95.3	19.7	75.6	18.0	−19.9	**<0.001**
SIT	41.1	13.3	45.0	12.5	31.7	10.5	−13.9	**<0.001**
TMTA	26.1	12.6	22.5	9.3	34.7	15.3	12.1	**<0.001**
TMTB	59.1	42	49.6	36.3	81.4	46.3	31.2	**0.002**
VFLT	36.4	12.9	.39.7	12.0	28.1	11.1	−12.1	**<0.001**
Composite Cognitive z-score	0.0	0.4	0.2	0.4	−0.4	0.4	−0.5	**<0.001**
Coping								
Active	19.8	4.0	20.6	3.4	17.6	4.8	−3.1	**0.010**
Palliative response	17.9	3.6	17.7	3.	18.5	4.1	0.9	0.429
Avoidance	16.5	4.0	15.9	3.7	18.2	4.2	2.4	**0.024**
Seeking social support	13.9	3.5	14.1	3.4	13.2	3.7	−0.8	0.375
Passive response	11.2	3.3	10.7	2.8	12.5	4.5	1.8	0.101
Expression of emotions	5.8	1.7	5.8	1.7	5.7	1.9	−0.1	0.921
Reassuring thoughts	12.1	2.9	12.1	2.9	12.3	3.0	0.3	0.708
FRSBE participant								
Apathy	25.5	9.6	24.3	8.9	28.8	10.4	4.5	0.052
Disinhibition	25.2	8.8	24.7	8.4	26.7	9.7	2.0	0.357
Executive	30.6	11.2	28.8	10.4	35.4	11.9	6.6	**0.015**
Total	81.4	27.9	77.7	26.4	90.9	30.1	13.2	0.053
FRSBE proxy								
Apathy	24.3	7.7	22.3	7.5	28.3	6.7	6.0	**0.018**
Disinhibition	23.9	6.9	23.2	8.4	25.4	7.4	2.2	0.370
Executive	31.4	10.7	29.5	1.4	35.2	9.1	5.7	0.098
Total	79.6	23	75.0	26.4	88.8	21.5	13.8	0.074
UHDRS								
TMS	9.7	11.6	3.9	5.3	24.0	10.3	20.3	**<0.001**
TFC occupation (%)	82/35 (70.1/29.9)		2.6	0.6	2.5	0.9	−0.1	0.425
DCL (pre-motor manifest/ motor manifest) (%)	84/33 (70.5/29.5)							
DCL-scores (0, 1, 2, 3, 4)			35/23/11/15		/33			

CAG: Cytosine Adenosine Guanine; CAP: CAG-Age product; DCL: Diagnostic Confidence Level; FRSBE: Frontal Behaviors Examination; SCNT: Stroop Color Naming Test; SDMT: Symbol Digits Modalities Test; SIT: Stroop Interference Test; SWRT: Stroop Word Reading Test; TFC: Total Functioning Capacity; TMS: Total Motor Score; TMT A & B: Trail Making Test A & B; UCL: Utrechtse Coping Lijst; VCFT: Verbal Category Fluency Test; VLFT: Verbal Letter Fluency Test. p-values < 0.05 are shown in bold to indicate statistical significance.

Motor manifest participants demonstrate more behavior consistent with anosognosia, as determined through expert judgment by (neuro)psychologists (χ^2^ = 7.32, p = 0.007) and the combined measure of anosognosia (χ^2^ = 8.07, p = 0.004). The other isolated assessments of anosognosia conducted through physician expert judgment, participant ratings, and actual performance, as well as participant ratings and proxy ratings, revealed no differences between pre-motor manifest and motor manifest participants. Moreover, the two groups had no significant distinctions regarding other coping domains or work outcomes (see Table 1).

Analysis of the FRSBE measures revealed that motor manifest participants rated themselves significantly higher on executive problems than pre-motor manifest participants (t = –2.53, p = 0.015). Slightly differently, proxies rated motor manifest participants as significantly more apathetic (t = –2.52, p = 0.018) than pre-motor manifest participants (Table 1). However, in paired-sample t-tests comparing FRSBE measures between proxies and participants for total scores, as well as scores for pre-motor manifest and motor manifest groups, only pre-motor manifest participants showed significant differences in self-evaluation across all domains compared to their respective proxies; i.e., in the domains of apathy self-evaluated 24.4 vs. 22.0 proxy-reported (t = –2.61, p = 0.015), self-evaluated 24.9 vs. 23.2 proxy-reported disinhibition (t = –2.6, p = 0.015), and self-evaluated 78.1 vs. 74.4 proxy-reported total scores (t = –2.65, p = 0.014). These three domains show that overall, participants evaluated themselves as having more frontal behaviors. The domain of executive functioning, however, showed an inverted evaluation: self-evaluated 28.9 vs. 29.2 proxy-reported (t = –1.84, p = 0.077).

### Operationalization of anosognosia

**Figure 1.**
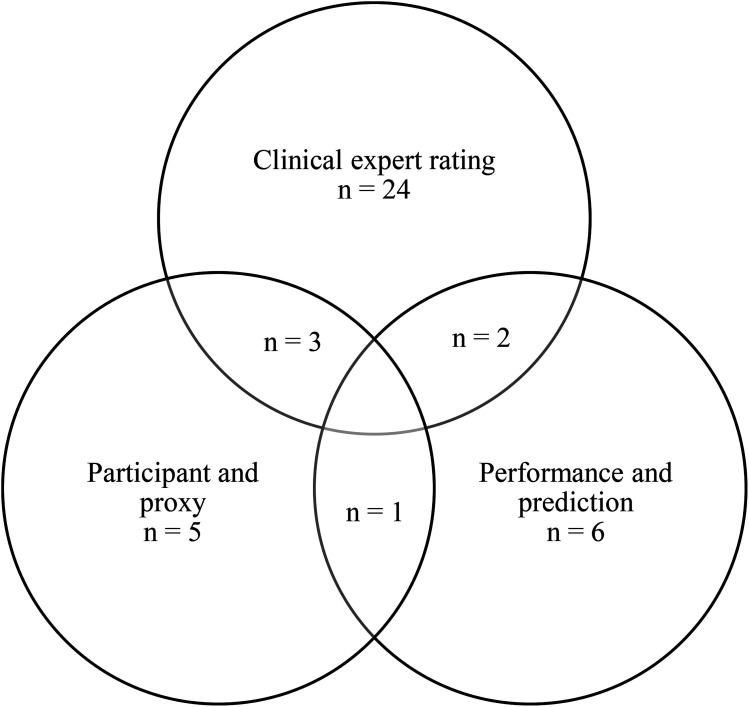
Venn diagram depicting the number of participants with anosognosia based on the different methods of determining anosognosia. Note: This Venn diagram visualizes the overlap in participants identified as having anosognosia based on three different approaches: (1) clinical expert rating (n = 24), which consisted of the ratings of physicians (n = 13), neuropsychologists (n = 17), (2) discrepancy between participant and proxy reports (n = 5), and (3) discrepancy between predicted and actual performance (n = 6). Participants could be classified via one or multiple methods. The overlapping areas illustrate cases where multiple methods agreed. In total, 30 individuals were classified as having anosognosia by at least one of the three approaches.

In total, 30 individuals had anosognosia or lacked awareness of any form (Figure 1). Most individuals were identified through clinical expert rating. The physicians identified 13 participants with anosognosia, whereas the (neuro)psychologists identified slightly more; n = 17, they were six times in agreement over anosognosia and 79 (67%) cases in agreement that there was no anosognosia. Ultimately, individuals classified as having anosognosia by any one or a combination of the three methods were chosen to comprise the combined anosognosia group (n = 30).

### Participants with vs. without anosognosia

We grouped participants without anosognosia and participants with anosognosia based on the combined anosognosia measure (Table 2). Participants without anosognosia in this sample were more often pre-motor manifest and had lower motor scores, had lower cag-repeat lengths (t = –2.48, p = 0.018), had a lower disease burden (t = –3.41, p = 0.001), scored better on cognitive measures (i.e., SDMT (t = 5.846, p < 0.001), VCFT (t = 3.372, p = 0.002), SCNT (t = 5.730, p < 0.001), SWRT (t = 4.418, p < 0.001), SIT (t = 4.750, p < 0.001), TMTA (t = –2.751, p = 0.010), TMTB (t = –2.495, p = 0.018), VFLT (t = 3.991, p < 0.001), moreover they were more likely to use an active coping response (t = 3.372, p = 0.002) (Table 2). They scored significantly lower on passive coping as compared to the participants with anosognosia (t = –2.352, p = 0.029). Participants without anosognosia rated themselves significantly lower and were rated lower by their proxies on all FRSBE domains as compared to participants with anosognosia (i.e., FRSBE participant; apathy (t = –3.034, p = 0.005), disinhibition (t = –3.204, p = 0.004), executive (t = –3.490, p = 0.002), total (t = –3.376, p = 0.002), and FRSBE proxy; disinhibition (t = –3.317, p = 0.003), executive (t = –2.113, p = 0.050, total (t = –2.640, p = 0.016)), except for apathy measured by proxies, which was not significantly different as compared to participants with anosognosia. No differences were found between the groups in work outcomes, sex, and age.

**Table 2.** Comparison of participants with anosognosia and without anosognosia.

	With anosognosia (N = 30)		Without anosognosia (N = 87)			
	Mean	SD	Mean	SD	Δ	p
Demographical						
Age	46.5	11.7	43.3	10.0	−3.2	0.185
CAG-repeat length	43.7	3.6	41.9	2.4	−1.8	**0.018**
Sex (f/m (%))	11/19 (36.7/63.3)		40/47 (46.0/54.0)			0.501
CAP-score	78.9	21.8	93.8	20.2	−14.9	0.**001**
Cognition						
SDMT	39.0	10.5	52.8	11.2	13.8	**<0.001**
VFCT	17.2	7.0	22.3	6.0	5.0	**0.002**
SCNT	55.1	12.3	71.7	15.0	16.5	**<0.001**
SWRT	75.8	18.0	94.0	20.3	18.2	**<0.001**
SIT	31.7	11.7	44.2	12.4	12.6	**<0.001**
TMTA	33.3	17.1	23.7	9.7	−9.5	**0.010**
TMTB	80.1	54.9	52.0	34.2	−28.0	**0.018**
VFLT	28.0	12.1	39.0	12.0	11.0	**<0.001**
Composite Cognitive z-score	−0.4	0.4	0.1	0.4	0.5	**<0.001**
Coping						
Active	17.3	3.2	20.5	4.0	3.2	**0.001**
Palliative response	17.8	4.2	17.9	3.5	0.2	0.884
Avoidance	17.9	4.4	16.1	3.8	−1.8	0.129
Seeking social support	13.6	3.7	13.9	3.4	0.3	0.801
Passive response	13.3	4.8	10.6	2.6	−2.7	**0.029**
Expression of emotions	6.1	2.0	5.7	1.7	−0.4	0.474
Reassuring thoughts	11.7	2.5	12.3	3.1	0.6	0.398
FRSBE participant						
Apathy	32.1	13.1	23.5	7.1	−8.6	**0.005**
Disinhibition	31.9	12.7	23.2	5.9	−8.8	**0.004**
Executive	38.9	14.1	28.0	8.8	−10.8	**0.002**
Total	102.9	38.6	74.7	19.6	−28.2	**0.002**
FRSBE proxy						
Apathy	27.8	8.5	22.8	7.0	−5.1	0.086
Disinhibition	28.6	5.6	21.8	6.4	−6.8	**0.003**
Executive	37.1	12.1	28.8	9.2	−8.3	**0.050**
Total	93.5	22.4	73.4	20.8	−20.1	**0.016**
UHDRS						
TMS	19.3	13.8	6.4	8.6	−12.8	**<0.001**
DCL/Premotor manifest/Motor manifest (%)	15/15 (50.0/50.0)		69/18 (79.3/20.7)			**0.005**
TFC occupation	2.5	0.7	2.6	0.7	0.1	0.662

CAG: Cytosine Adenosine Guanine; FRSBE: Frontal Behaviors Examination; SCNT: Stroop Color Naming Test; SDMT: Symbol Digits Modalities Test; SIT: Stroop Interference Test; SWRT: Stroop Word Reading Test; TFC: Total Functioning Capacity; TMS: Total Motor Score; TMS: Total Motor Score; TMT A & B: Trail Making Test A & B; UCL: Utrechtse Coping Lijst; VCFT: Verbal Category Fluency Test; VLFT: Verbal Letter Fluency Test. p-values < 0.05 are shown in bold to indicate statistical significance.

### Coping styles

In this study, we were interested in avoidant coping and how it relates to other coping styles. Focusing on the current sample's overall scores, we saw approximately 24% missing values in the UCL. Here, most missing values were introduced by male participants (Supplemental Figure 2). Comparing coping scores to UCL norms revealed that the sample scored 100% high/very high in passive coping (>80 percentile), followed by 44.4% in comforting coping. For avoidant coping, this was only 11%. Males tended to score higher in avoidant coping (5.9% vs. 2.6%), whereas females showed strength in social coping (9.4% vs. 3.4%).

**Figure 2.**
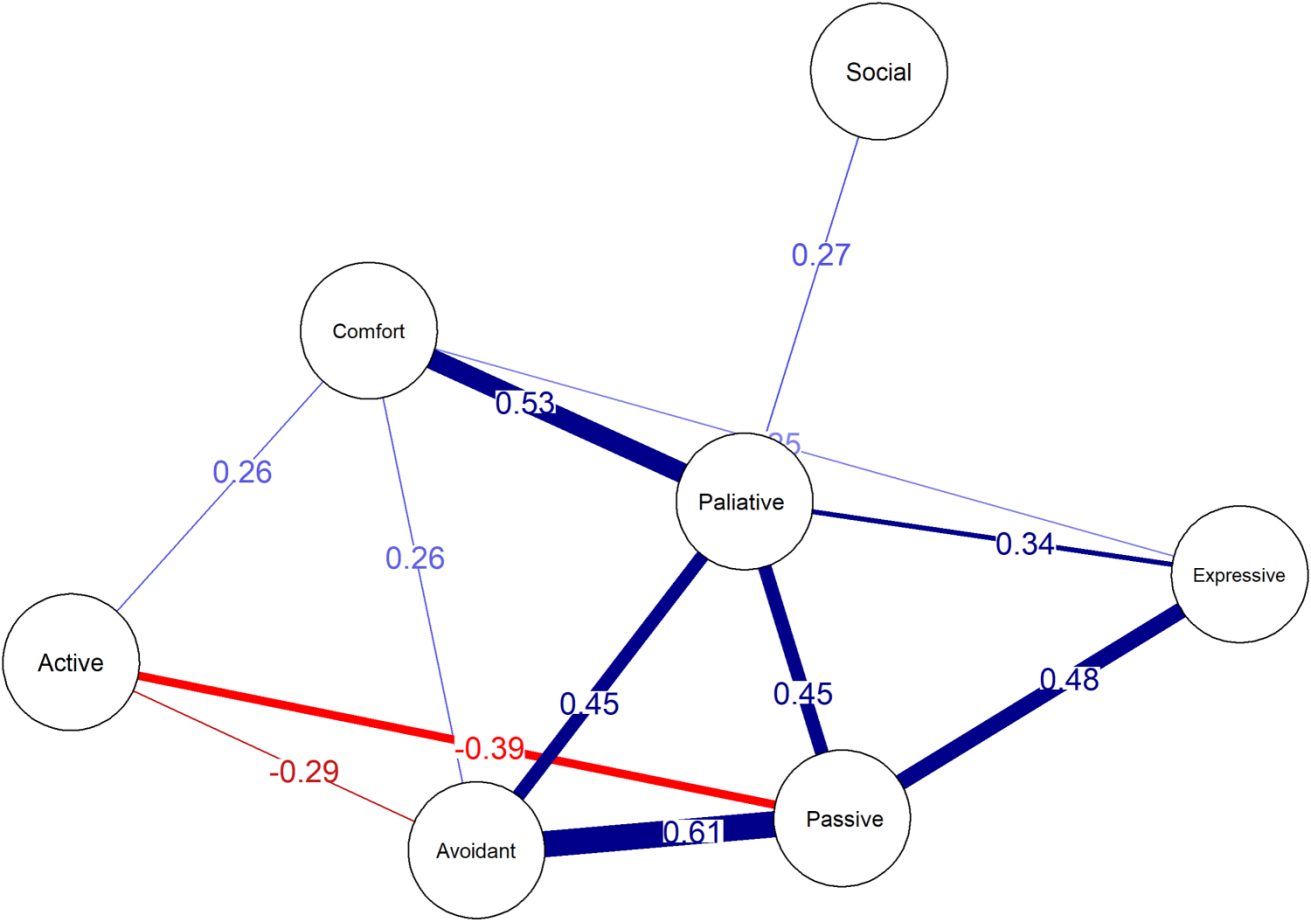
Network analysis of coping styles. Note: The direction of the correlations is indicated by the sign of the correlation coefficient (positive or negative). The threshold for visualizing edges was set at r = 0.1.

We decided to conduct a network analysis of the coping strategies asked about in the UCL. As shown in Figure 2, the coping network is well-balanced, with a few nodes having more centrality than others, i.e., palliative responses. A relatively strong positive correlation existed between avoidant and passive coping and comfort and palliative responses. Moderately strong positive correlations were observed between expressive and passive coping, palliative and avoidant coping, and palliative and passive responses. A moderately negative correlation was found between active and passive coping styles. There was very little correlation between social coping and other coping styles. Passive coping was strongly correlated to apathy as measured by the FRSBE (r = 0.695, p < 0.001).

### Anosognosia vs. avoidance and work

**Figure 3.**
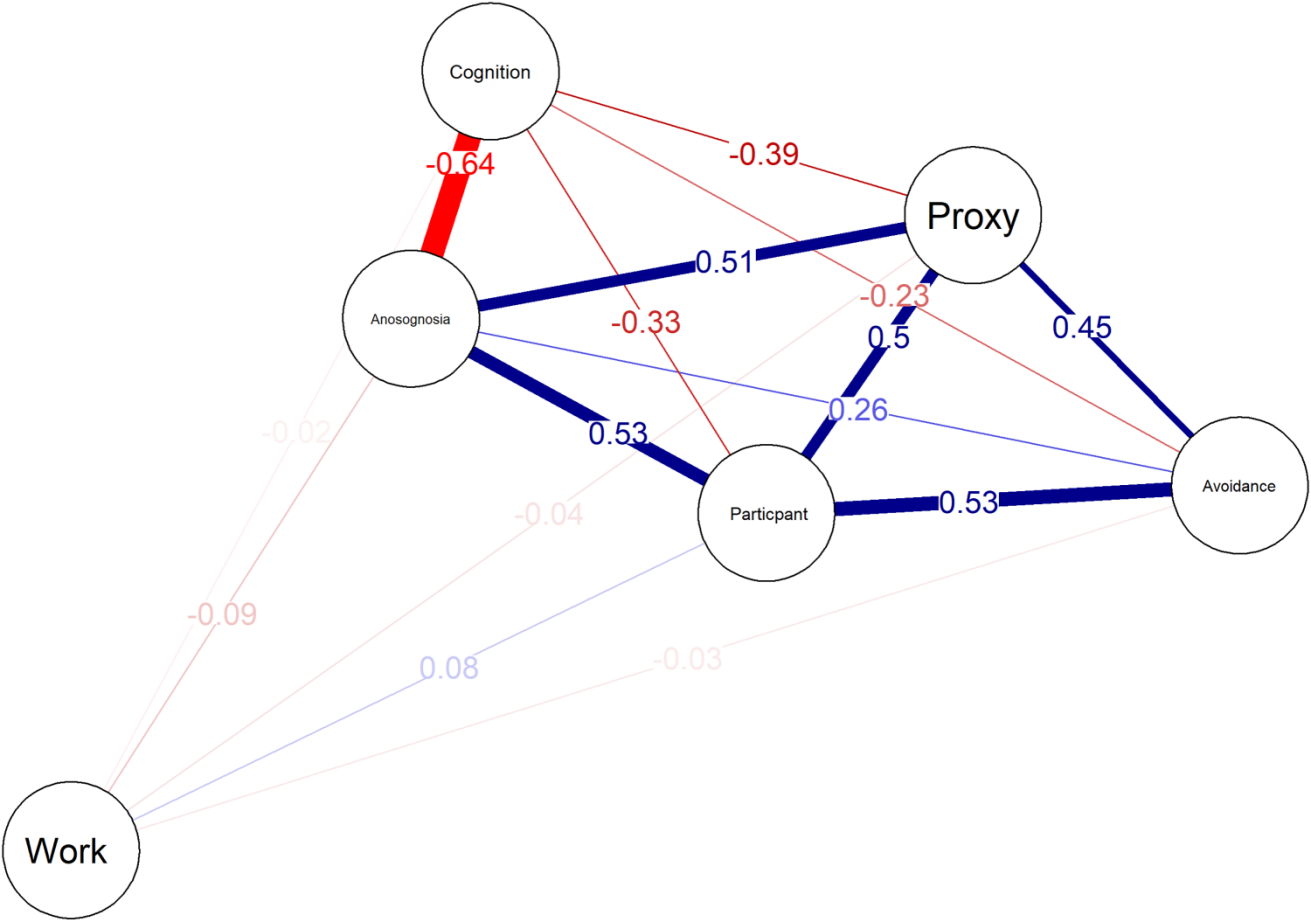
Network analysis of anosognosia, avoidance, and work. Positive correlations are in blue, and negative correlations are in red. The threshold for correlation visualization was set at r = 0.1. The Proxy and Participant nodes are FRSBE measures, the Cognition node is the composite cognitive z-score, the Anosognosia node is the combined anosognosia measure, and the Avoidance node is the avoidance coping measured by the UCL.

Univariate logistic regression analyses were conducted to evaluate the individual predictive influence of each variable on work outcome. The results showed that none of the predictors had statistically significant effects (all p-values > 0.05). A multivariate logistic regression model was also performed, which included multiple predictors such as anosognosia, avoidant coping, and various cognitive and behavioral measures. However, the model yielded no significant findings (all p-values > 0.05), suggesting that the combination of these variables did not significantly predict work outcome. The overall model did not show a strong fit, as evidenced by the low residual deviance and high AIC (35.132). Pearson's correlation between avoidant coping and anosognosia produced no significant results (r = 0.04, p = 0.740). We, therefore, chose to perform a network analysis again (Figure 3). The ‘Work’ node did not correlate to other nodes with a r > 0,1. The strongest negative correlation was found between composite cognitive z-scores and anosognosia. The lower the cognitive score, the bigger the chance a participant has anosognosia. Moreover, participant and proxy FRSBE ratings were moderately negatively correlated to cognitive performance since higher scores on the FRSBE mean more frontal problems. Avoidance was positively correlated to the FRSBE measures. The higher the avoidant coping style, the more people reported frontal problems. The association between the FRSBE measures (the nodes Participant and Proxy) and anosognosia was characterized by a direct correlation, indicating that as the FRSBE scores increased, indicating more severe frontal impairments, the incidence of anosognosia also rose.

## Discussion

Avoidant coping or lack of awareness can strain relationships of Huntington's Disease Expanded Gene Carriers, affecting not only family but also professional ties. Employees who deny their inability to perform the job or are unaware of impairments may face situations where employers wrongly attribute their shortcomings to factors other than HD, for example, lack of motivation and underachievement. This study investigated the potential influence of anosognosia and avoidant coping on working capacity in the early stages of HD, thereby examining their relationships with other coping styles and clinical characteristics. While our investigation did not find significant direct effects between anosognosia, coping styles, and work outcomes, it is crucial to view these results within the larger context of HD research. These findings underscore the limited direct impact of anosognosia and coping styles on work outcomes, particularly in the early stages of HD. Nonetheless, their importance remains as they highlight the complexity of these factors and their interactions in the broader scope of HD research.

### Anosognosia

Anosognosia is an elusive construct that is difficult to grasp; simply asking individuals if they are avoiding the issue at hand or if they are unaware of their disease is paradoxical and ineffective. Therefore, we have utilized Ruijter et al.'s spectrum of methods to determine (a suspected) anosognosia.^25^ Results indicate that a quarter of our pre-motor manifest to early motor manifest cohort either had or were suspected of anosognosia, which further underlines that anosognosia can play a role early in HD.^6^ Notably, the various techniques selected different participants as having anosognosia, and the group designated by multiple methods seemed to be marginal. The combined anosognosia measure and the judgment of a (neuro)psychologist revealed a distinct difference in the amount of anosognosia when comparing pre-motor manifest and motor manifest participants. When we contrasted physicians’ assessments with those of (neuro)psychologists, we observed that they did not always align, with psychologists being more inclined to label someone as having anosognosia. Importantly, anosognosia in Huntington's disease is a multi-faceted phenomenon; the same individual may be unaware of certain symptoms (e.g., chorea) while retaining awareness of other impairments, such as cognitive decline or apathy, highlighting that it does not have to be generalized across all symptom domains.^7,9^ Therefore, these discrepancies may have arisen due to the multi-faceted nature of anosognosia, as well as variations in the time spent with participants and differences in the topics discussed.

Overall, participants without anosognosia performed better on all clinical characteristics. In this sample, anosognosia was most associated with cognitive performance, which aligns with earlier studies,^9,10,29^ especially for frontal cognitive performance.^30^ Interestingly enough, participants and proxies could provide insights into their cognitive performance equally, which is not in line with previous studies.^31^ Moreover, participants with anosognosia rated their frontal behaviors as more problematic than participants without anosognosia, paradoxically implying that they are aware. This paradox is inconsistent with research suggesting that anosognosia can result in the underreporting of cognitive and behavioral changes, such as apathy, even though these changes may be quite apparent to others.^8^ A significant difference between the current study and Cleret de Langavant et al. is the cognitive domain studied; we focus primarily on executive dysfunction, whereas their study focuses on memory deficits and the disease stage. Although memory problems are a commonly heard complaint in HD patients, the actual existence of memory impairments is somewhat of a neuropsychological debate, especially in the early stages of the disease. Memory issues often seem to result from executive dysfunction. Early-stage HD patients show slowed processing of information, but their memory functions normally, whereas later-stage patients experience more severe slowing and impairments in acquiring and retrieving information.^32^ There is no “gold standard” tool for screening cognitive dysfunction in HD, and memory scales are not overly recommended.^33^ Therefore, memory tests were not included in our study. This limitation means we cannot definitively state that memory impairments did not influence the coping styles observed. Moreover, another explanation might be that participants in our study are simply too far away from clinical onset, and therefore exhibit fewer frontal behaviors.

Our findings showed that anosognosia was not only present in the late stages of HD but also in some pre-motor manifest cases. This supports the notion that anosognosia can emerge early in the disease, even before full motor manifestation, highlighting the need for early detection.^9^ Identifying anosognosia early can improve how we manage work-related challenges, support caregivers and employers, and plan for future care needs. Additionally, early recognition of anosognosia enables healthcare providers to implement support strategies, such as cognitive and behavioral therapies, to better address the needs of individuals at earlier stages of the disease. This finding is consistent with previous studies,^9^ emphasizing the need for caretakers and clinicians to recognize anosognosia before late-stage HD. Moreover, the presence of anosognosia in the pre-motor manifest group could reflect the emergence of early neuropsychiatric symptoms, aligning with findings from Duff et al. (2010).^30^ This underscores that frontal and other neuropsychiatric changes can precede overt motor manifestations, indicating the importance of nuanced interpretation and early intervention.

### Coping

The *Utrechtse Coping Lijst* (UCL) was used in the context of a work-related study, therefore conclusions drawn from its results should always be viewed in that context. Avoidant coping, a style of dealing with stress by suppressing or avoiding problems, is less prevalent than anosognosia. Avoidance is mainly correlated with the self and proxy's views of the frontal behaviors of the participant. It is more often seen in motor manifest than pre-motor manifest participants. Earlier research states that palliative, hope, and self-monitoring are HD's most utilized coping strategies.^34^ In our study, the most frequently used coping style was passive coping, where people do not face problems by taking direct action. This coping style seemed to be closely related to apathy, which is an often-encountered psychiatric characteristic of HD. This means individuals may disengage from active problem-solving and instead exhibit a withdrawn or resigned approach, which is characteristic of apathy.^9^ This finding is consistent with research that links anosognosia and avoidant coping with reduced mental flexibility and attention, which may exacerbate apathy. Nevertheless, their association should be a topic for future research. Overall, the participants in this study did not often seek social comfort. People tend to express their problems but do not take an active role and avoid forming solutions.

### Anosognosia and avoidance

The study found that avoidant coping was positively correlated with anosognosia, which paradoxically suggests that individuals who tend to avoid or suppress their problems may be more likely to lack awareness of their impairments. Moreover, anosognosia and avoidant coping were negatively related to cognitive decline, suggesting that individuals with poorer cognitive function may be more likely to experience anosognosia and utilize avoidant coping strategies. This finding is underlined by the fact that in this study, both anosognosia and avoidant coping strategies were more often seen in motor manifest rather than pre-motor manifest participants. The findings might indicate a misattribution of avoidant coping to behaviors associated with anosognosia.

### Differences between patient and caregiver ratings

The striking difference in ratings between patients and caregivers on the FrSBe was only found in pre-motor manifest individuals. This observation can be explained by the fact that, in the early, pre-manifest stages of HD, subtle changes in frontal behavior may be more noticeable to the patients themselves because they still retain self-awareness of minor cognitive or behavioral shifts. This is in line with previous studies,^30^ which suggest that individuals in pre-manifest HD may be particularly attuned to these subtle changes in their own behaviors, and therefore, may overestimate their problems. Conversely, some individuals in the manifest stage may underestimate their difficulties, a phenomenon that – if clinically significant and persistent – can be classified as anosognosia, as discussed in the study by Duff et al. Moreover, caregivers do not perceive these subtle changes.

As the disease progresses into the motor manifest stage, patients’ self-awareness often declines, which likely leads to a reduction in the discrepancy between self-reports and caregiver assessments, as suggested by Cleret de Langavant et al.^31^ However, not all manifest-stage patients experience anosognosia, suggesting that other factors may also contribute to this convergence (Table 1).

One possible explanation is that behavioral symptoms become more pronounced and objectively noticeable, making patient and caregiver assessments more aligned. Additionally, while caregivers may gradually adapt to behavioral changes over time, our data show that caregivers of patients with anosognosia report higher behavioral problem scores than caregivers of patients without anosognosia (Table 2). This may lead to higher caregivers’ ratings on the FrSBe, but not (yet) significantly higher than the patient's self-ratings, because the investigated cohort was still in an early motor-manifest stage.

### Potential limitations

One limitation of our study is the dichotomization of the cohort into individuals with and without anosognosia. Including a dedicated anosognosia, assessment would have provided a more nuanced understanding by allowing us to quantify the degree of anosognosia in our participants. This, in turn, would have enabled a more precise examination of its relationship with the clinical characteristics of HD, frontal behaviors as measured by the FrSBe, and coping styles. Secondly, the narrow inclusion criteria requiring participants to be currently working or have worked in the last two years may restrict the generalizability of our findings. This focus was intended to ensure that participants had recent and relevant work experience, which might provide a clearer link between anosognosia, coping styles, and work capacity. However, this narrow criterion also potentially limits the statistical power and generalizability of our results, as it excludes individuals who might be in different stages of work-related functioning. The cohort specifically focuses on primarily pre-motor manifest and early stage HD participants, underlining that a clinical motor manifestation of the disease does not automatically lead to employment disability.

Thirdly, the use of the Total Functional Capacity (TFC) scale, specifically its occupation item, as a measure of work capacity may have limitations. Relying on a single item from the TFC to measure work capacity may oversimplify the complexity of assessing an individual's ability to work. Although this approach facilitates the analysis and comparison of results, it may not capture the full spectrum of work-related challenges faced by individuals with Huntington's. Despite this, the TFC's established reliability in HD research offers a validated framework for evaluating functional abilities.

## Conclusion

This study sheds light on the intricate dynamics of avoidance and awareness in Huntington Disease Expanded Gene Carriers. Our study revealed that these factors do not directly influence working capacity in early HD stages. Anosognosia showed a higher prevalence in motor manifest participants and a strong correlation with cognitive decline. Avoidant coping, less prevalent than anosognosia, was linked to frontal behaviors and was also more commonly observed in motor manifest participants. Strikingly, the study found a positive association between avoidant coping and anosognosia, suggesting potential misattribution of avoidant coping behaviors consistent with anosognosia.. This misattribution could be due to a misunderstanding of the nature of avoidant coping behaviors, which may be perceived as a lack of awareness rather than a coping strategy. Additionally, this study emphasizes the role of cognitive function in these constructs. These findings underscore the need for early recognition of anosognosia, as well as for avoidant and passive coping, not solely in late-stage HD.

## Supplemental Material

sj-docx-1-hun-10.1177_18796397251349114 - Supplemental material for Anosognosia and avoidant coping do not impact work in early Huntington's disease Supplemental material, sj-docx-1-hun-10.1177_18796397251349114 for Anosognosia and avoidant coping do not impact work in early Huntington's disease by Kasper Frederik van der Zwaan, Raymund AC Roos and Susanne T de Bot in Journal of Huntington's Disease
