# Supplementary material for: Anosognosia and avoidant coping do not impact work in early Huntington's disease
Source: J Huntingtons Dis. 2025 Jun 11;14(2):179–90. doi: 10.1177/18796397251349114 (PMC12231776; doi:10.1177/18796397251349114)

**Supplemental Material**

**Anosognosia and avoidant coping do not impact work in early Huntington’s disease**

| **Supplemental Table 1.** Full demography of the sample. | | | | |  |
| --- | --- | --- | --- | --- | --- |
|  | Min | Median | Max | Missing | Valid |
| Demographical |  |  |  |  |  |
| Age | 21 | 44 | 64 | 0 | 117 |
| CAG-repeat length | 36 | 42 | 56 | 0 | 117 |
| sex (f/m (%)) |  |  |  | 0 | 117 |
| physician anosognosia (y/n (%)) |  |  |  | 13 | 104 |
| psychologist anosognosia (y/n (%)) |  |  |  | 5 | 112 |
| Cognition |  |  |  |  |  |
| SDMT | 20 | 50 | 77 | 8 | 109 |
| VFCT | 7 | 21 | 40 | 8 | 109 |
| SCNT | 32 | 69 | 108 | 8 | 109 |
| SWRT | 12 | 92 | 141 | 9 | 108 |
| SIT | 5 | 41 | 82 | 9 | 108 |
| TMTA | 11 | 24 | 79 | 8 | 109 |
| TMTB | 3 | 48 | 240 | 10 | 107 |
| VFLT | 7 | 36 | 71 | 13 | 104 |
| Composite Cognitive z-score | -1.0 | 0.04 | 1.35 | 16 | 101 |
| Coping |  |  |  |  |  |
| Active | 9 | 20 | 27 | 28 | 89 |
| Palliative response | 10 | 18 | 26 | 28 | 89 |
| Avoidance | 8 | 16 | 29 | 28 | 89 |
| Seeking social support | 7 | 13 | 23 | 31 | 86 |
| Passive response | 7 | 11 | 28 | 28 | 89 |
| Expression of emotions | 3 | 6 | 12 | 29 | 88 |
| Reassuring thoughts | 5 | 12 | 18 | 27 | 90 |
| FRSBE participant |  |  |  |  |  |
| Apathy | 6 | 24 | 61 |  |  |
| Disinhibition | 8 | 24 | 58 |  |  |
| Executive | 8 | 28 | 69 |  |  |
| Total | 23 | 76 | 182 | 20 | 97 |
| FRSBE proxy |  |  |  |  |  |
| Apathy | 7 | 24 | 39 |  |  |
| Disinhibition | 9 | 23 | 38 |  |  |
| Executive | 8 | 32 | 51 |  |  |
| Total | 24 | 79 | 118 | 78 | 39 |
| UHDRS |  |  |  |  |  |
| TMS | 0 | 4 | 46 | 3 | 114 |
| DCL (premanifest/manifest) | 0 | 2 | 4 | 0 | 117 |
| TFC occupation (normal/reduced) | 0 | 3 | 3 | 0 | 117 |

CAG: Cytosine Adenosine Guanine; DCL: Diagnostic Confidence Level; FRSBE: Frontal Behaviors Examination; SCNT: Stroop Color Naming Test; SDMT: Symbol Digits Modalities Test; SIT: Stroop Interference Test; SWRT: Stroop Word Reading Test; TFC: Total Functioning Capacity; TMS: Total Motor Score; TMT A & B: Trail Making Test A & B; UCL: Utrechtse Coping Lijst; VCFT: Verbal Category Fluency Test; VLFT: Verbal Letter Fluency Test

**Supplemental Figure 1.** The formula used to calculate a composite cognitive z-score.

$$Composite Cogntive z-score=\left( \frac{\begin{aligned} \sum(\left( \frac{x^{SDMT}-\mu^{SDMT}}{\sigma^{SDMT}} \right)+\left( \frac{x^{VCFT}-\mu^{VFCT}}{\sigma^{VFCT}} \right)+\left( \frac{x^{SCNT}-\mu^{SCNT}}{\sigma^{SCNT}} \right)+\left( \frac{x^{SWRT}-\mu^{SWRT}}{\sigma^{SWRT}} \right)+ \\ \left( \frac{x^{SIT}-\mu^{SIT}}{\sigma^{SIT}} \right)+\left( \frac{x^{TMTA}-\mu^{TMTA}}{\sigma^{TMTA}} \right)+\left( \frac{x^{TMTB}-\mu^{TMTB}}{\sigma^{TMTB}} \right)+\left( \frac{x^{VFLT}-\mu^{VFLT}}{\sigma^{VFLT}} \right)) \end{aligned}}{8} \right)$$

The z-scores of all participants' cognitive measures are calculated by dividing the score on the test minus the group's mean by the standard deviation. The sum of all z-scores is divided by the number of cognitive assessments.

**Supplemental Figure 2.** Normative stratification of the UCL coping domains grouped per sex.


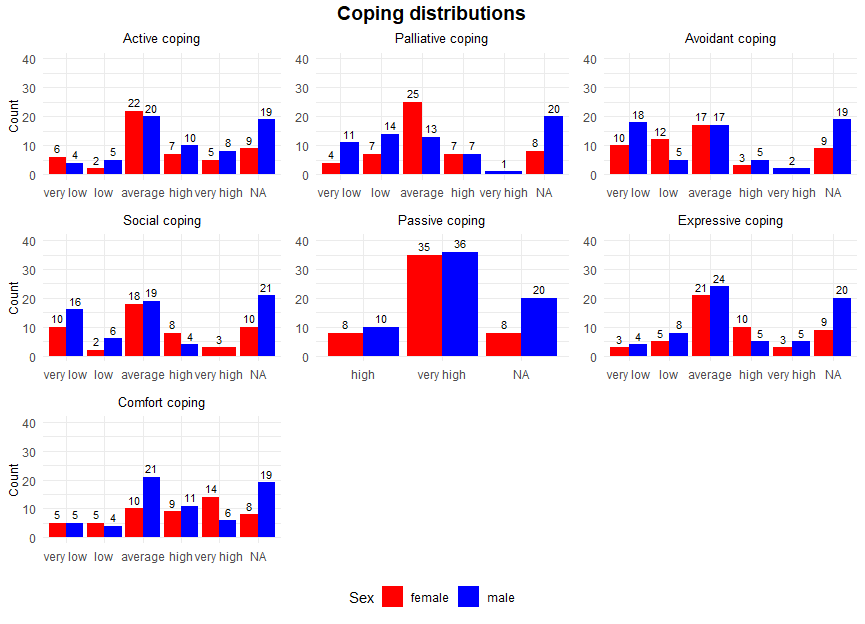

Supplement: sj-docx-1-hun-10.1177_18796397251349114 - Supplemental material for Anosognosia and avoidant coping do not impact work in early Huntington's disease [file sj-docx-1-hun-10.1177_18796397251349114.docx]
